# Supplementary material for: Identification and Functional Analysis of Two UGT84 Glycosyltransferases in Flavonoid Biosynthesis of Carthamus tinctorius
Source: Plants (Basel). 2025 Oct 9;14(19):3112. doi: 10.3390/plants14193112 (PMC12525976; doi:10.3390/plants14193112)
Supplement: Supplementary file 1 [file plants-14-03112-s001.zip › plants-3863393-supplementary.pdf]

Supporting Information

Identification and functional analysis of two UGT84 glycosyltransferases in flavonoid biosynthesis of *Carthamus tinctorius*

Chaoxiang Ren [1,2], Jinxin Guo [1,2], Siyu Liu [1,2], Bin Xian [1,2], Yuhang Li [1,2], Changyan Yang [1,2], Cheng Peng [1,2,], Jin Pei [1,2,*] and Jiang Chen [1,2,*]

1 State Key Laboratory of Southwestern Chinese Medicine Resources, Chengdu University of Traditional Chinese Medicine, Chengdu 611137, China
2 College of Pharmacy, Chengdu University of Traditional Chinese Medicine, Chengdu 611137, China
* Correspondence: pengchengchengdu@126.com (C.P.); peixjin@163.com (J.P.); janshen1986@163.com (J.C.)

Content

Figure S1. Induction and western blot analysis of recombinant UGT proteins........................ 2

Figure S2. Amino acid sequences alignment of enzymes in UGT84 subfamily. 3

Table S1. Bioinformatics analysis of 2 candidate UGTs... 4

Table S2. Primers for UGTs cloning 5

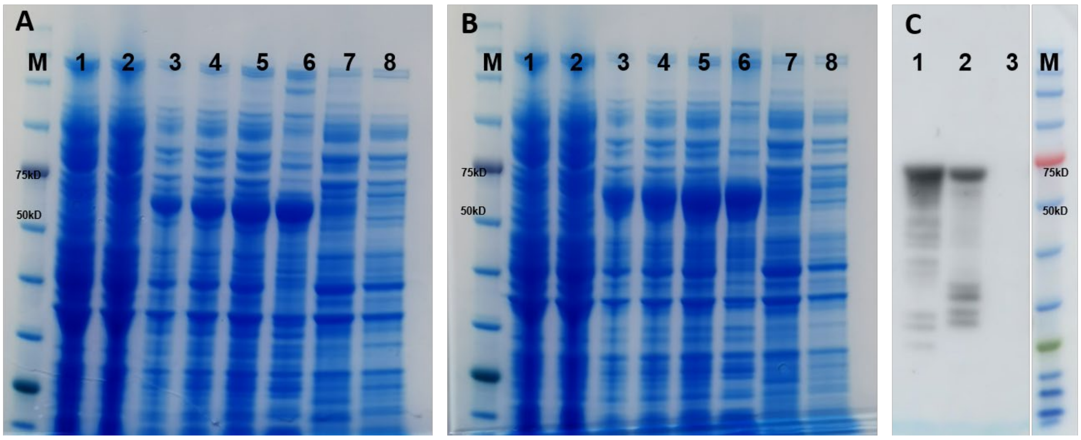

Figure S1. Expression and western blot analysis of recombinant UGT proteins. (A) expressions of UGT84A28 recombinant proteins; M is the protein marker; Lane 1-2, total protein after transferring UGT84A28 without IPTG induction; lanes 3-5, total protein after transferring UGT84A28 with 3, 9, and 16 h IPTG induction; lane 6, supernatant after ultrasonic fragmentation using the 16 h cultured bacteria with IPTG; lane 7, precipitation after ultrasonic fragmentation using the 16 h cultured bacteria with IPTG; lane 8, total protein after transferring empty vector with 16 h IPTG induction; (B) expressions of UGT84B3 recombinant proteins; M is the protein marker; Lane 1-2, total protein after transferring UGT84B3 without IPTG induction; lanes 3-5, total protein after transferring UGT84B3 with 3, 9, and 16 h IPTG induction; lane 6, supernatant after ultrasonic fragmentation using the 16 h cultured bacteria with IPTG; lane 7, precipitation after ultrasonic fragmentation using the 16 h cultured bacteria with IPTG; lane 8, total protein after transferring empty vector with 16 h IPTG induction; (C) western blot analysis of recombinant UGT proteins, 1 was unpurified UGT84A28 recombinant proteins, 2 was unpurified UGT84B3 recombinant proteins, 3 was negative control with empty vector, M was protein maker.

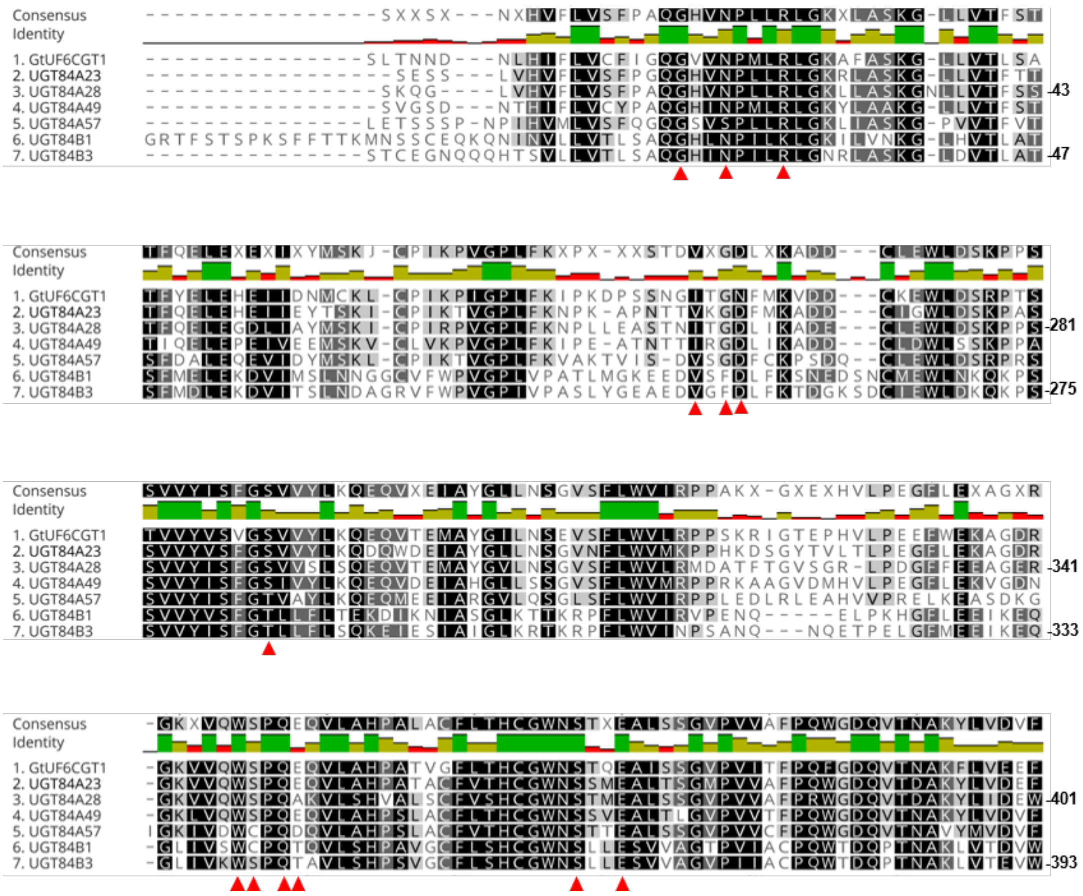

Figure S2. Amino acid sequences alignment of enzymes in UGT84 subfamily.

The sites marked with red triangles were the residues mentioned in this study.

Table S1. Bioinformatics analysis of 2 candidate UGTs

Gene name	Length	Amino acid	MW (kD)	pI	Formula	Extinction coefficients	Aliphatic index
UGT84A28	1512	503	55.9	5.71	$C_{2482}H_{3903}N_{673}O_{740}S_{27}$	69160	84.10
UGT84B3	1413	470	52.1	5.58	$C_{2333}H_{3645}N_{621}O_{695}S_{18}$	68910	87.09

Table S2. Primers for UGTs cloning

Primers name	Sequences (5’-3’)
UGT84A28-F	ATGGCTTCCAAACAAGGCCT
UGT84A28-R	TCAATTGGGCACCGGACA
UGT84B3-F	ATGGATTCGACTTGTGAAGGA
UGT84B3-R	CTATTTGGAATTACATGAAG
pET32-UGT84A28-F	CGAGCTCCGTCGACAAGCTTATGGCTTCCAAACAAGG CCT
pET32-UGT84A28-R	GAGTGCGGCCGCAAGCTTTCAATTGGGCACCGGACA
pET32-UGT84B3-F	CGAGCTCCGTCGACAAGCTTATGGATTCGACTTGTGAA GGA
pET32-UGT84B3-R	GAGTGCGGCCGCAAGCTTCTATTTGGAATTACATGAAG
pDonor-UGT84A28-F	GGGGACAAGTTTGTACAAAAAAGCAGGCTTAATGGCT TCCAAACAAGGCCT
pDonor-UGT84A28-R	GGGGACCACTTTGTACAAGAAAGCTGGGTATCAATTG GGCACCGGACA
pDonor-UGT84B3-F	GGGGACAAGTTTGTACAAAAAAGCAGGCTTAATGGAT TCGACTTGTGAAGGA
pDonor-UGT84B3-R	GGGGACCACTTTGTACAAGAAAGCTGGGTACTATTTGG AATTACATGAAG
